# Odontogenic Cutaneous Fistula-Induced Submandibular Abscess in a Dog: A Rare Presentation

**DOI:** 10.3390/vetsci12111071

**Published:** 2025-11-07

**Authors:** Jong-Mu An, Won-Jong Lee, Dae-Hyun Kim, Seong Mok Jeong, Yoon-Ho Roh, Dongbin Lee, Chang-Hwan Moon

**Affiliations:** 1 Department of Veterinary Surgery, College of Veterinary Medicine, Gyeongsang National University, 501, Jinjudae-ro, Jinju 52828, Republic of Korea; radish94@naver.com (J.-M.A.); yoonhoroh@gnu.ac.kr (Y.-H.R.); dlee@gnu.ac.kr (D.L.); 2 Department of Veterinary Surgery, College of Veterinary Medicine, Chungnam National University, 99, Daehak-ro, Yuseong-gu, Daejeon 34134, Republic of Korea; wjl03ssaaa@naver.com (W.-J.L.); vet1982@cnu.ac.kr (D.-H.K.); jsmok@cnu.ac.kr (S.M.J.)

**Keywords:** odontogenic cutaneous fistula, periapical abscess, submandibular abscess, dog

## Abstract

**Simple Summary:**

Odontogenic cutaneous fistulas usually develop from maxillary periapical infections that drain externally through the facial skin. Mandibular odontogenic cutaneous fistulas are not common because of the thick mandibular cortex and limited drainage pathways. We describe the case of a 14-year-old male mixed-breed dog that had a chronic periapical infection of the right mandibular first molar tooth that penetrated the mandibular bone and extended into the submandibular soft tissues, which formed a draining fistulous tract. The lesion was initially misdiagnosed as a salivary gland cyst. However, computed tomography confirmed a fistulous connection between the mandibular apex and the subcutaneous lesion. Surgical removal of the affected tooth and tract achieved complete resolution. This case emphasizes that mandibular odontogenic cutaneous fistulas can mimic submandibular abscesses and highlights the importance of using cross-sectional imaging and individualized surgical management for accurate diagnosis and treatment.

**Abstract:**

Odontogenic cutaneous fistulas (OCFs) are relatively uncommon in veterinary patients. They are typically caused by chronic periapical infections of the maxillary teeth. Mandibular OCFs that extend through the cortical bone into submandibular soft tissues are extremely rare. This report describes the case of a 14-year-old male mixed-breed dog that presented with a submandibular cutaneous lesion initially misdiagnosed as a salivary mucocele. OCFs are frequently misdiagnosed because atypical presentations may lack obvious intraoral abnormalities and mimic salivary gland disease, lymphadenopathy, or cutaneous neoplasia. Computed tomography (CT) revealed a periapical lesion associated with the right mandibular first molar, cortical bone lysis, and extension into the adjacent submandibular tissues, which formed an external fistulous tract. Surgical management included extraction of the affected tooth, resection of the fistulous tract, and excision of the associated lymph nodes, which resulted in a complete and uneventful resolution of the fistula. At the 3-month follow-up, the patient remained clinically stable without evidence of recurrence. This case underscores the clinical pearl that odontogenic origins should always be considered in the differential diagnosis of submandibular or cervical cutaneous lesions and that cross-sectional imaging modalities, such as CT, are indispensable for confirming anatomical continuity and guiding surgical planning.

## 1. Introduction

Odontogenic cutaneous fistulas (OCFs) are uncommon sequelae of chronic periapical infections, most often secondary to pulpal necrosis caused by trauma, pulpitis, or advanced periodontal disease [1,2]. OCFs are most frequently associated with maxillary teeth, particularly canines and first molars, owing to their thin cortical bone and proximity to facial skin. Most reported cases involve maxillary lesions draining to the periorbital or facial region [3,4,5]. In contrast, mandibular OCFs are extremely rare owing to the thicker mandibular cortex and limited drainage pathways, with only a few cases documented in the literature [6,7]. In such cases, the infection penetrates the dense mandibular cortex and extends into the submandibular soft tissues, creating a diagnostic challenge.

Atypical presentations without overt intraoral abnormalities may lead to misdiagnoses such as salivary gland disease, lymphadenopathy, or cutaneous neoplasia [4,5]. To aid clinicians, the principal differential diagnoses for submandibular or cervical cutaneous swellings are summarized in Table 1 [8].

This diagnostic challenge is particularly pronounced in geriatric patients, where chronic dental disease may be masked by age-related soft tissue atrophy, concurrent systemic illness, or owners attributing appetite or weight changes to normal aging. Consequently, the odontogenic origins may be overlooked, resulting in delayed or inappropriate treatment.

Advanced imaging techniques, particularly computed tomography (CT), are pivotal for indicating the continuity between periapical lesions and cutaneous tracts. Unlike conventional dental radiography, CT provides three-dimensional visualization of periapical pathology, cortical bone defects, and soft tissue extension [8,9]. While plain dental radiographs may be sufficient for early or localized lesions, CT offers superior delineation of cortical lysis, fistula continuity, and soft tissue spread, making it indispensable in advanced or atypical cases [10,11,12]. Recent studies further demonstrated that thin-slice and cone beam CT provide more precise dentoalveolar assessment and improve diagnostic accuracy compared to conventional radiography [10,11]. When an odontogenic origin is confirmed, the definitive treatment involves extraction or endodontic therapy of the affected tooth, with or without surgical resection of the tract [1,4,7].

Herein, we describe a rare case of mandibular OCF in a geriatric dog initially misdiagnosed as a salivary gland mass. Cross-sectional imaging was critical for establishing the diagnosis and surgical plan. This case underscores the importance of recognizing and including dental diseases in a differential diagnosis for submandibular cutaneous lesions, especially when clinical presentation is atypical.

## 2. Case Description

**Figure 1:**
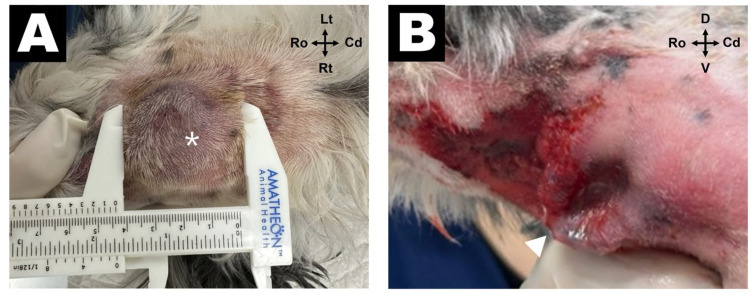
Gross appearance of the right submandibular lesion. (**A**) Initial presentation showing a firm, erythematous, subcutaneous mass (asterisk) in the right ventral mandibular region at initial presentation. (**B**) One day after admission, the lesion spontaneously ruptured, producing purulent discharge and a centrally ulcerated skin defect (arrowhead), consistent with a cutaneous fistulous tract.

A 14-year-old castrated male mixed-breed dog was referred to the Veterinary Medical Teaching Hospital of Gyeongsang National University for evaluation of a subcutaneous mass located in the right submandibular region, which was initially suspected to be a salivary gland tumor by a veterinarian at a local hospital. The mass was incidentally discovered 1 day prior to presentation. The dog had recently become reluctant to chew dry kibbles and preferred soft foods. On physical examination, a subcutaneous soft tissue mass measuring approximately 3 cm in diameter was palpated at the level of the right mandibular angle, with mild local pain observed upon palpation (Figure 1A).

**Figure 2:**
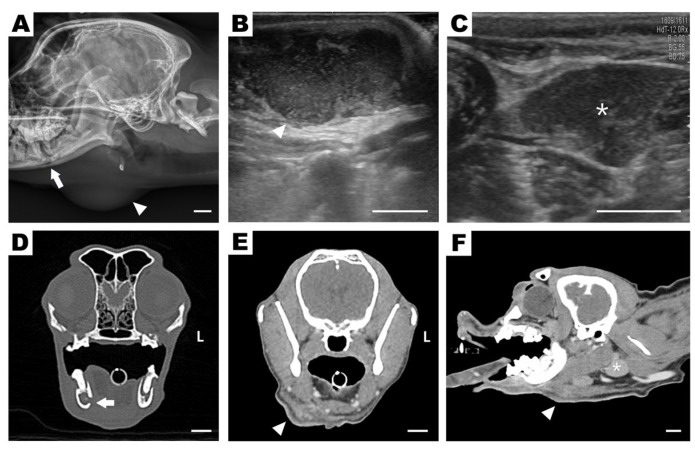
Preoperative diagnostic imaging: radiography (**A**), ultrasonography (**B**,**C**), and computed tomography (CT) (**D**–**F**). (**A**) Lateral skull radiograph obtained before abscess rupture, showing alveolar bone lysis around the right mandibular first molar (409) (white arrow) and a soft tissue opacity ventral to the mandibular angle (arrowhead), suggestive of a submandibular lesion. (**B**) Longitudinal ultrasound image of the submandibular region showing a cavitary, hypoechoic lesion (arrowhead). (**C**) Transverse ultrasound of the right mandibular salivary gland with preserved echotexture and no ductal dilation (asterisk). (**D**) Post-rupture transverse CT image at the level of the right mandibular first molar showing cortical bone discontinuity (arrow) due to periapical lysis. (**E**,**F**) CT images showing an enhancing, cavitary tract breaching the mandibular cortex and extending into the submandibular tissue (arrowhead), consistent with an external draining fistula. The right mandibular salivary gland appears unaffected (asterisk). Scale bars = 10 mm.

Lateral skull radiographs revealed alveolar bone lysis surrounding the right mandibular first molar (409) and an area of increased soft tissue opacity ventral to the angular process (Figure 2A). Ultrasonography revealed a cavitary, hypoechoic lesion in the subcutaneous region (Figure 2B), whereas a normal mandibular salivary gland echotexture without signs of ductal dilatation was observed (Figure 2C). A tubular hypoechoic structure cranial to the lesion was also observed, suggesting either a dilated salivary duct or a fistulous tract. Based on these findings, differential diagnoses included abscess, ruptured salivary duct, and salivary mucocele. On the day after admission, the subcutaneous lesion ruptured spontaneously, discharging purulent material externally and revealing a draining tract on the skin surface (Figure 1B). Further assessment using CT revealed discontinuity of the mandibular cortical bone adjacent to the mesial root of tooth 409, indicating infectious bone destruction (Figure 2D). A rim-enhancing, ill-defined cavitary lesion extending from the periapical area into the ipsilateral submandibular subcutaneous tissue was observed (Figure 2E,F), strongly suggestive of a draining fistulous tract of odontogenic origin.

Collectively, the imaging findings supported the diagnosis of OCF originating from a periapical abscess of the right mandibular first molar. The lesion penetrated the mandibular cortex and extended through soft tissues into the submandibular skin. Complete blood count (CBC) and serum biochemistry performed during hospitalization revealed no clinically significant abnormalities. Bacterial culture and antimicrobial susceptibility testing were performed on samples obtained from the mandibular abscess and fistulous tract. No aerobic bacterial growth was detected after 48 h of incubation, and antimicrobial susceptibility testing was therefore not available. Although aerobic culture and susceptibility testing yielded no bacterial growth, amoxicillin–clavulanate was administered empirically, given its broad-spectrum activity against common oral anaerobes and Gram-positive organisms typically implicated in odontogenic infections. Therefore, surgical treatment was planned.

The patient was premedicated with Midazolam (0.2 mg/kg, IV; Bukwang Midazolam Inj., Bukwang Pharm, Seoul, Republic of Korea) as a sedative, Butorphanol tartrate (0.2 mg/kg, IV; Butophan Inj., Myungmoon Pharm, Seoul, Republic of Korea) as an analgesic, Cefazolin (25 mg/kg, IV; Cefozol Inj., Hankook Korus Pharm, Chuncheon, Republic of Korea) as a prophylactic antibiotic, and Famotidine (1 mg/kg, IV; Gaster^®^ Inj., Donga ST, Seoul, Republic of Korea) as a gastrointestinal protectant.

Anesthesia was induced with Alfaxalone (2 mg/kg, IV; Alfaxan^®^ Multidose, Zoetis, Parsippany-Troy Hill, NJ, USA) and maintained with Isoflurane (Ifran^®^ Liq., Hana Pharm, Seoul, Republic of Korea) in 100% oxygen.

During surgery, analgesia was maintained using a constant rate infusion (CRI) of Tramadol (1 mg/kg/h; Tramadol HCl Inj., Shinpoong Pharm, Seoul, Republic of Korea), Lidocaine (2 mg/kg/h; Lidocaine HCl Hydrate Inj. 1%, Dai Han Pharm, Seoul, Republic of Korea), and Ketamine (0.6 mg/kg/h; Ketamine HCl Inj., Huons, Seongnam, Republic of Korea).

Additionally, an inferior alveolar nerve block was performed prior to extraction of tooth 409 using 0.5% Bupivacaine (Myungmoon Bupivacaine^®^, Myungmoon Pharm, Seoul, Republic of Korea) and 2% Lidocaine (Lidocaine HCl Injection^®^, Dai Han Pharm, Seoul, Republic of Korea) to provide immediate and prolonged local desensitization.

**Figure 3:**
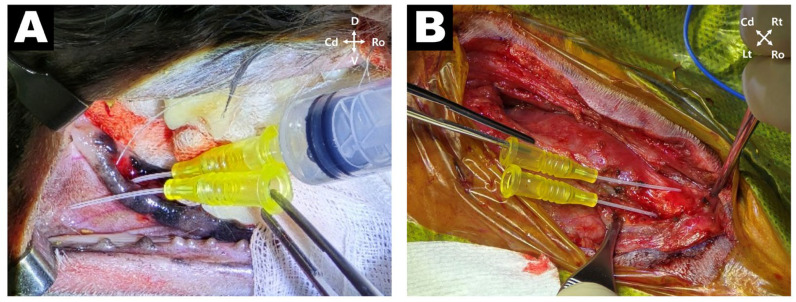
Intraoperative findings. (**A**) Extraction of the right mandibular first molar (409): a 24-gauge catheter is inserted into the alveolar socket, confirming an intraosseous fistulous tract. (**B**) Following debridement of the submandibular lesion, a 24-gauge catheter is inserted from the subcutaneous tract, and continuity with the intraoral fistula is confirmed.

The patient underwent tooth extraction and surgical excision of the associated inflammatory lesion under general anesthesia. The dog was positioned in left lateral recumbency; intraoral scaling and hemisecting extraction of tooth 409 were performed using a high-speed burr and dental elevators. Following extraction, a 24-gauge catheter was inserted into the alveolar socket to evaluate fistula tract patency. The catheter was passed through the periapical lesion and emerged into the subcutaneous space, confirming anatomical continuity with the cutaneous fistula (Figure 3A). Hemostasis was achieved using epinephrine-soaked gauze, and the exposed socket was closed with a gingival flap using 4-0 Monocryl in a simple interrupted pattern. The patient was then repositioned in dorsal recumbency with the neck extended in a humanoid position. A skin incision was made over the submandibular lesion to approach the subcutaneous tract. The purulent exudate and inflamed fibrous tissue were thoroughly debrided. Two enlarged mandibular lymph nodes adjacent to the lesion were identified, resected, and submitted for histopathological analysis. To reconfirm tract continuity, a 24-gauge catheter was inserted into the center of the submandibular lesion and successfully passed into the mandibular bone, delineating the full path of the OCF (Figure 3B). The wound was flushed with 200 mL of warm sterile saline, and a Barovac drain was placed. The subcutaneous tissues were closed using 4-0 PDS in a simple interrupted pattern, and the skin was closed using 4-0 nylon in an interlocking pattern.

Postoperative analgesia was provided using a transdermal buprenorphine patch (Norspan patch^®^ 20 µg/h; Mundipharma Korea, Seoul, Republic of Korea), applied immediately after surgery. In addition, the patient received amoxicillin–clavulanate (Clavamox^®^, Zoetis, Parsippany, NJ, USA) at 20 mg/kg PO q12 h for 14 days and meloxicam (Metacam^®^, Boehringer Ingelheim, Ingelheim, Germany) at 0.1 mg/kg PO q24 h for 5 days. A closed suction drain (Barovac^®^, SS200L, 8 Fr, Sewoon Medical, Seoul, Republic of Korea) was placed intraoperatively to manage dead space and serosanguinous discharge. The drain was removed on postoperative day (POD) 3 (27 June 2025), with minimal fluid output.

**Figure 4:**
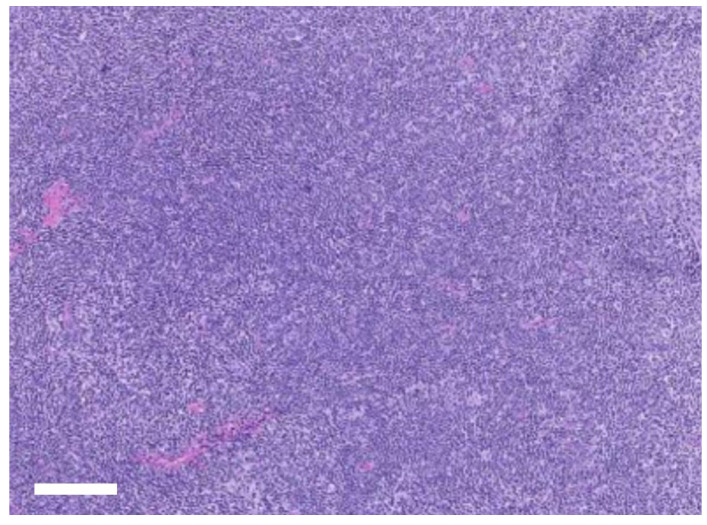
Histopathology of the excised right mandibular lymph node showing diffuse lymphoid hyperplasia with preserved follicular polarity and tangible body macrophages (H&E, ×100). Scale bar = 100 µm.

Histopathological examination of the excised right mandibular lymph node revealed diffuse lymphoid hyperplasia (Figure 4), consistent with chronic nonspecific antigenic stimulation and supporting a reactive change rather than neoplasia. The resected lesion also showed chronic inflammatory changes with fibrosis and neutrophilic infiltration, confirming the diagnosis of chronic odontogenic infection associated with cutaneous fistula formation. At the 7-day recheck, the surgical wounds were stable, with no swelling, discharge, or dehiscence, and intraoral healing was satisfactory. The dog maintained a good appetite and activity after extraction. At 2 weeks, sutures were removed without complication, and both intraoral and cutaneous sites had healed well. By 4 weeks, complete resolution of the cutaneous fistula was confirmed, with no pain on palpation and full return to normal feeding and behavior. At the 3-month follow-up, the surgical sites remained stable, with no recurrence of fistula, lymphadenopathy, or inflammation. The owner reported sustained improvements in appetite, comfort, and overall activity levels, indicating a successful outcome.

## 3. Discussion

In the present case, the tract originating from the right mandibular first molar (409) penetrated the cortical bone and drained externally through the submandibular skin. The atypical location and absence of overt oral symptoms initially led to the misdiagnosis of a salivary gland lesion. These diagnostic challenges are consistent with previous reports in which mandibular OCFs were mistaken for sialadenitis, reactive lymphadenopathy, or soft tissue neoplasms [7,8,10].

Mandibular OCFs remain exceptionally rare, which may be attributable to the thicker mandibular cortex and limited drainage pathways compared to the maxilla. These anatomical factors likely reduce the likelihood of fistula formation, thereby explaining why most reported cases originate from maxillary teeth [4,6].

Advanced imaging was decisive for achieving the correct diagnosis. Conventional radiography provided limited information, whereas CT revealed the periapical lesion, cortical lysis, and fistulous tract continuity. Although intraoral dental radiographs are generally recommended as the first-line modality, CT was prioritized in this case because the patient was referred with a presumptive salivary gland disease. CT not only excluded salivary gland involvement but also delineated the odontogenic tract with greater precision, compensating for the absence of intraoral dental radiographs [12]. Recent studies further emphasize the superior diagnostic accuracy of thin-slice and cone beam CT compared to conventional radiography for dentoalveolar evaluation [9,10,11].

The standard treatment for OCF involves eliminating the source of infection, usually through extraction or endodontic therapy. In many cases, the fistulous tract resolves after the primary dental lesion is removed [1,2]. However, in this case, the infection extended into the submandibular tissues, with associated fibrosis and reactive lymphadenopathy, necessitating a dual surgical approach: intraoral extraction of tooth 409 and extraoral excision of the tract and lymph nodes. This combined surgical strategy was tailored to the anatomical extent of the lesion and highlights the need for individualized interventions in advanced cases.

Histopathology of the excised mandibular lymph node supported a diagnosis of reactive change rather than neoplasia. This finding demonstrated that lymphadenectomy was not only therapeutic in reducing local inflammatory burden but also diagnostically valuable, allowing exclusion of neoplastic disease and confirming chronic antigenic stimulation as the underlying process. By integrating histopathological evaluation into the surgical approach, diagnostic certainty was achieved, thereby justifying the dual intraoral and extraoral intervention [12,13,14].

OCFs are typically polymicrobial infections involving mixed oral anaerobes (e.g., *Actinomyces* spp., *Fusobacterium* spp., and *Prevotella* spp.) and Gram-positive cocci. Although aerobic culture in this case showed no bacterial growth after 2 days of incubation, these organisms are well documented in canine periapical infections. Amoxicillin–clavulanate was chosen empirically because it provides broad coverage against both common oral anaerobes and β-lactamase–producing organisms. Recent veterinary dentistry surveys have confirmed its role as a widely used first-line antimicrobial in small-animal dental infections, consistent with antimicrobial stewardship principles [15,16,17].

This case also illustrates the potential for gravity-dependent spread of mandibular infections in geriatric patients with reduced soft tissue support [18]. Even when cutaneous lesions appear distant from the oral cavity, odontogenic causes should be considered. Regular dental evaluations, including dental radiography, are essential to prevent severe sequelae, such as OCF, particularly in older dogs. In this patient, a history of dental disease and lack of routine oral care emphasizes the importance of owner education and preventive canine dentistry [19].

Epidemiologically, OCFs are rare in veterinary patients, with only sporadic cases reported, most often from maxillary teeth [3,4,5,6]. In contrast, odontogenic sinus tracts are relatively common in human dentistry, largely due to the higher prevalence of untreated periapical infections [4]. In companion animals, subtle or absent intraoral signs often delay recognition, whereas routine dental care in humans facilitates earlier detection. This contrast underscores the clinical importance of OCFs in veterinary practice, particularly when they mimic other cervical or submandibular diseases.

This case highlights the diagnostic challenges of atypical OCF, the critical role of CT in establishing anatomical continuity, and the value of tailoring surgical strategies according to the extent and chronicity of the lesion. By documenting a mandibular OCF requiring both extraction and lymphadenectomy, this report extends the current literature, which has largely focused on maxillary presentations.

This case report has two main limitations. First, because the lesion had already ruptured before surgery, preoperative bacterial culture could not be performed. Although a postoperative culture of the excised tissue was submitted, it yielded no growth, which necessitated the use of empiric amoxicillin–clavulanate. Second, as this is a single case report, the findings cannot be generalized, and further studies with larger case numbers are needed to validate these observations.

## 4. Conclusions

This report documents a rare mandibular OCF requiring both intraoral extraction and extraoral lymphadenectomy. Unlike typical maxillary OCFs, the mandibular presentation posed diagnostic and therapeutic challenges. The case emphasizes that successful management of OCF depends on tailoring surgical strategies to the anatomical extent and chronicity of the lesion and on considering odontogenic disease as a differential diagnosis for submandibular cutaneous lesions.

## Figures and Tables

**Table 1:** Differential diagnoses for submandibular or cervical cutaneous lesions in dogs.

Category	Examples
Salivary disorders	Salivary mucocele, Sialadenitis
Lymphatic disorders	Reactive lymphadenopathy, Lymphadenitis
Infectious/Inflammatory	Bacterial abscess, Fungal granuloma
Neoplastic	Salivary gland tumors, Lymphoma, Other skin tumor

## Data Availability

The original contributions presented in this study are included in the article. Further inquiries can be directed to the corresponding author.

## Abbreviations

The following abbreviations are used in this manuscript:

CT	computed tomography
OCF	odontogenic cutaneous fistula
